# Report of the online meeting of the 7th Asian Conference on Environmental Mutagens (ACEM), China, November 5-6, 2022

**DOI:** 10.1186/s41021-023-00263-8

**Published:** 2023-02-07

**Authors:** Rui Zhang, Wen Chen

**Affiliations:** Guangdong Provincial Key Laboratory of Food, Nutrition and Health, Department of Toxicology, School of Public Health, Sun Yat-sen University, Guangzhou, 510080 China

**Keywords:** Asian Association of Environmental Mutagen Societies (AAEMS), Asian Conference on Environmental Mutagens (ACEM), Chinese Environmental Mutagen Society (CEMS), Qingdao

## Abstract

The 7th Asian Conference on Environmental Mutagens (ACEM 2022) was held online from November 5–6, 2022. However, the 19th Chinese Environmental Mutagen Society Meeting was postponed due to the pandemic prevention policies of COVID-19 and the time will be announced later. In total, 467 participants from 8 countries, including China, Japan, Korea, Philippines, etc. participated in the virtual conference. Eight keynote speakers and 16 lecturers in 2 symposia made their speeches online on topics aligned with the theme “The Impact of Global Change on Asian Environment and Genomic Health”. More than 270 abstracts were submitted in this conference. We sincerely appreciate the efforts of all the participants, organizers, and members from Asian Association of Environmental Mutagen Societies (AAEMS). ACEM 2022 was a success and provided an excellent platform for exchanging the latest developments and stimulating scientific collaboration in the Asia-Pacific region as well as other parts of the world.

The economic strength in the Asia-Pacific region has been rising in the last two decades. In parallel, the Asia-Pacific region consisting of different culture, customs, and economic levels, is facing diverse environmental and health problems. The Asian Association of Environmental Mutagen Societies (AAEMS) was established in 2005 when the International Conference on Environmental Mutagens (ICEM) in San Francisco was held. Eight member countries, including China, Korea, Japan, Philippines, Thailand, India, Iran, and Australia have joined AAEMS and provided great support to this conference. The 7th Asian Conference on Environmental Mutagens (ACEM 2022) was hosted by AAEMS, the Chinese Environmental Mutagen Society (CEMS) and organized by Qingdao University and Sun Yat-sen University.

ACEM 2022 was held online from November 5 -6, 2022.

## Opening remarks

**Fig. 1:**
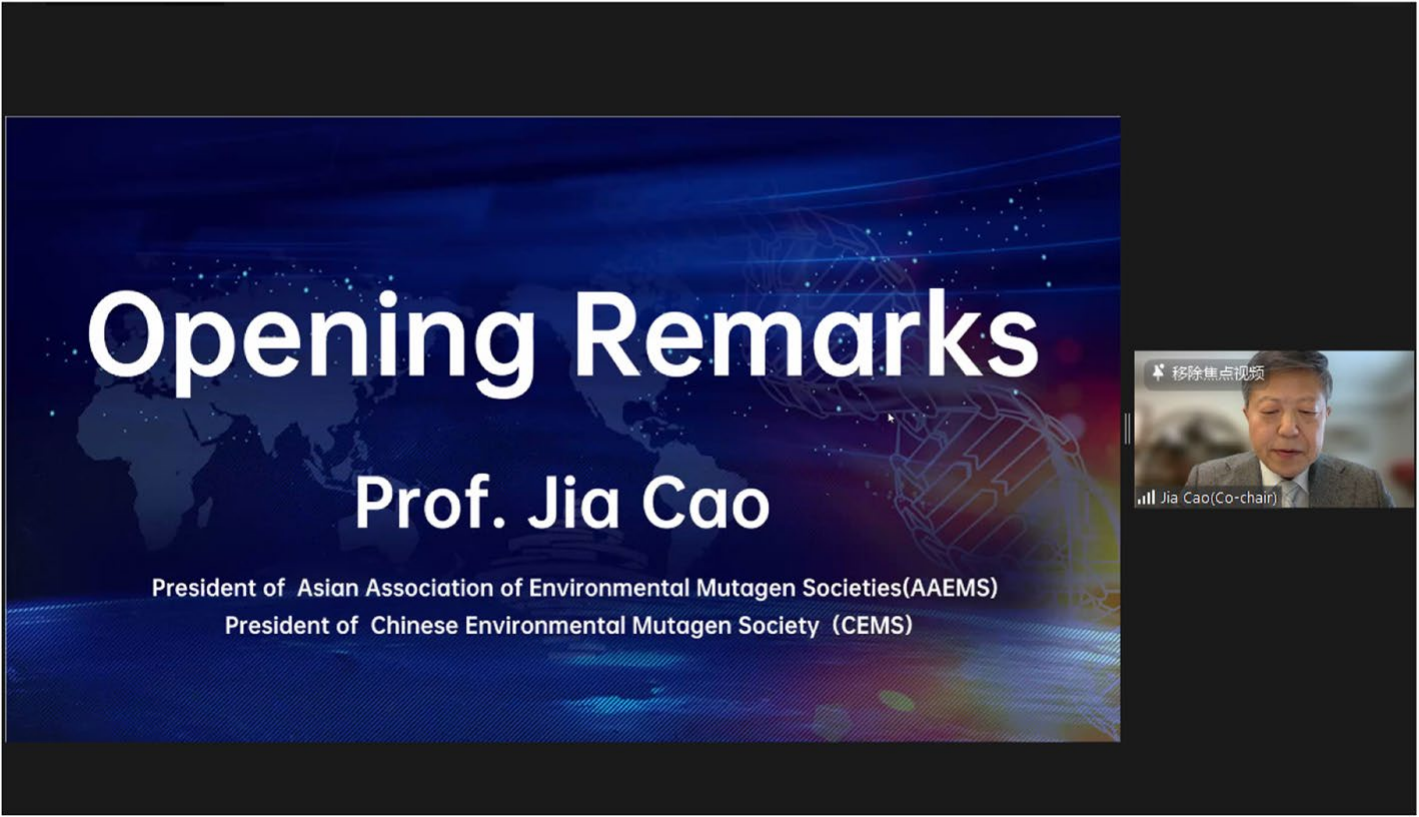
Opening remarks by Prof. Jia Cao

The conference was inaugurated by Prof. Jia Cao, the chairman of the ACEM 2022, the president of the AAEMS and CEMS (Fig. 1). Prof. Cao first warmly welcomed all invited participants at the ACEM 2022. The ACEM 2022 aimed to promote fundamental and translational research on environmental mutagens and genomic health in Asia and the Pacific Rim, and to promote close cooperation within the region. This was the first time that ACEM was held online. Prof. Cao thanked all the members for participating in the ACEM 2022.

## Keynote lectures

At this conference, 8 cutting-edged keynote lectures were given by invited eminent and internationally recognized experts from different regions, including Prof. Thomas Hartung from the United States, Dr. Federica Madia from France, Academician Aristides M. Tsatsakis and Dr. Elisavet Renieri from Greece, Prof. Masami Yamada from Japan, Prof. Keon Wook Kang from Korea, and Academician Dongxin Lin, Prof. Wei Huang and Prof. Hailin Wang from China. Their presentations covered a broad range of advanced fields of environmental health sciences, such as application of novel techniques in risk assessment, development of genome research, advances in cancer research and epigenetic mechanisms of adverse health outcomes induced by environmental hazards.

Prof. Hartung, Dr. Madia, Academician Tsatsakis, Dr. Elisavet and Prof. Huang delivered valuable presentations describing advances in risk assessment of xenobiotics from different dimensions. Prof. Hartung depicted a broad overview of the application of artificial intelligence in identifying potential carcinogens. Based on her work in the International Agency for Research on Cancer, Dr. Madia summarized the general principles and procedures for scientific review and evaluation to identify carcinogens to humans. Taking real life simulation into consideration, Academician Tsatsakis and Dr. Renieri introduced the establishment and application of integrative approaches to perform regulatory risk assessment. Prof. Huang systematically reviewed the efficacy of short-term intervention against air pollution related to personal exposure levels, applying her experience in collaboration with the World Health Organization. In addition, Academician Lin, Prof. Yamada, Prof. Kang, and Prof. Wang provided their remarkable insights into novel approaches in revealing toxicity mechanisms. Academician Lin delivered an introduction of their incredible work regarding depicting the body map of somatic mutations and clonal expansions from the same individuals. As the president of The Japanese Environmental Mutagen and Genome Society (JEMS), Prof. Yamada introduced three recent achievements in genome research in Japan and discussed her vision for the future of genome research in Asia. Based on his innovative work, Prof. Kang described his work on circulating small extracellular vesicles on aggressive cancer cells, which could be considered as a potential targeting strategy to handle aggressive cancer. Last but not least, with his strong background in ultrasensitive analytical technologies for detecting carcinogenic DNA adducts and DNA-repair proteins interactions, Prof. Wang demonstrated his world-wide seminal work on establishing a linkage among chemical exposure, DNA hydroxymethylation, and tumor-associated proliferation.

## Symposia

During the two-day conference, 16 outstanding scientists, including 1 from the United States, 3 from Japan, 1 from Korea and 11 from China, were invited to give oral presentations in symposia. The symposia mainly focused on several topics listed below.

- DNA damage and repair, and environmental mutagenesis and genomic instability
- Toxicity mechanisms of environmental chemicals
- Applications of new technologies and approaches in risk assessment of environmental chemicals
- Exposure science and exposome

Among these topics, lectures covered a wide range of pollutants and carcinogens, including heavy metals, ambient pollutants, aryl organophosphate flame retardants, perfluorooctane sulfonate, and polycyclic aromatic hydrocarbons. Novel approaches including multi-omics, quantitative structure-activity relationship (QSAR), adverse outcome pathway, physiologically based pharmacokinetics (PBPK) models and exposome were applied in these innovative studies.

All the oral presentations in symposia were encouraging and inspiring. The lecturers shared their insights with the attendees, offered them opportunities for connecting with the experts and disseminated the latest scientific development on environmental mutagens in the Asia-Pacific region. These high-quality oral presentations provoked significant exchange of ideas among both young scholars and experts.

## AAEMS executive committee members meeting

**Fig. 2:**
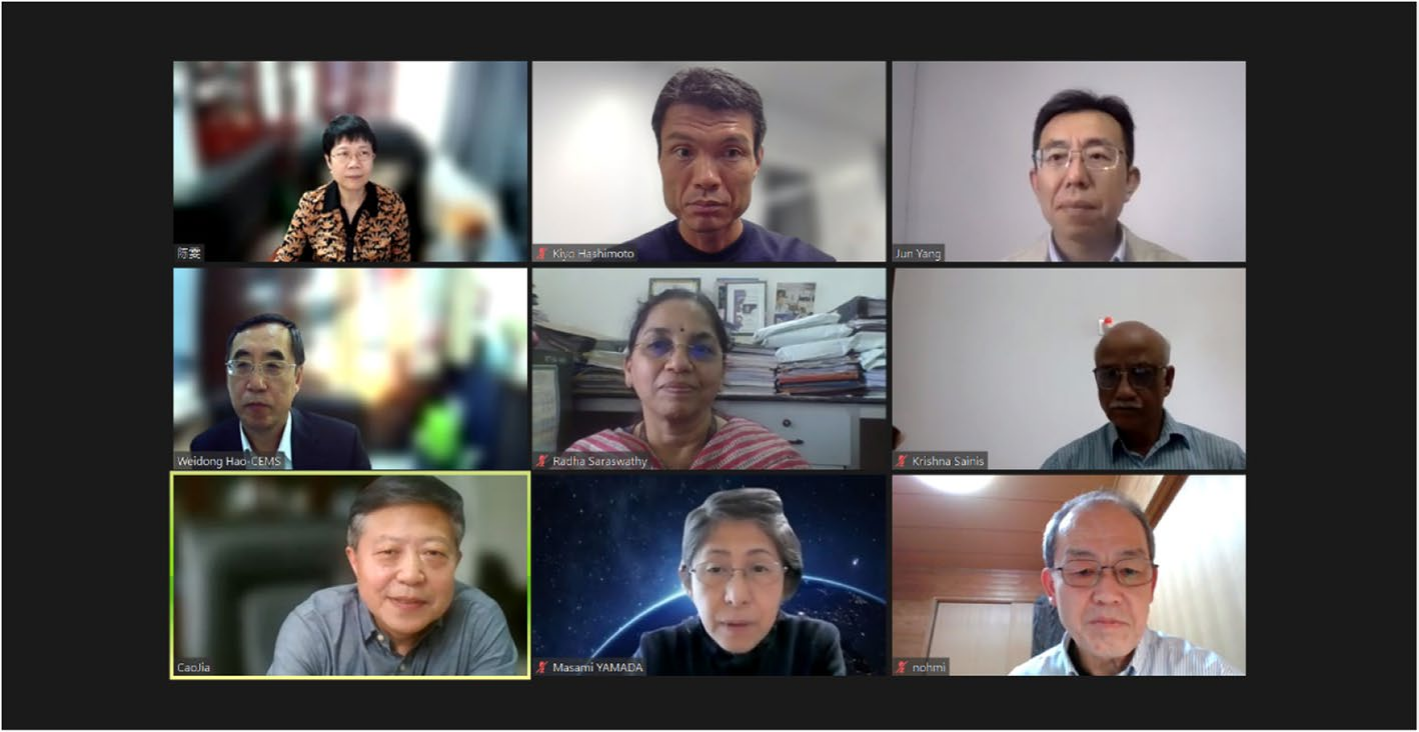
10th AAEMS executive committee members meeting

The 10th executive committee meeting of the AAEMS was held online in the afternoon of November 3, 2022 (Fig. 2). Members of the AAEMS from eight countries attended the online meeting and gathered together to discuss the working schedule of the AAEMS in the next 3 years.

## Poster session

In addition to the invited lectures, delegates from the Asia-Pacific region submitted more than 270 abstracts in both English and Chinese for symposia and poster sessions from domestic and overseas regions. All the abstracts will be collected in the Abstracts of ACEM 2022 and published in conjunction with the 19th Chinese Environmental Mutagen Society Meeting in electronic version. The poster session will be on-site at the 19th Chinese Environmental Mutagen Society Meeting.

## Conclusion

The ACEM 2022 was a success, with tremendous contributions and support from the experts, scientists, and organizers. In total, 467 delegates from China, Japan, Korea, Singapore, USA, France, Greece and Philippines participated in the online two-day conference. In the closing remarks, Prof. Cao congratulated the host of the conference and greatly thanked the scientists, Environmental Mutagen Society (EMS) representatives from the Asia-Pacific region and participants for their participation. At the 19th Chinese Environmental Mutagen Society Meeting, distinguished oral presentations and posters will be awarded in order to encourage their outstanding work. Experts and colleagues participating in the upcoming 19th Chinese Environmental Mutagen Society Meeting in Qingdao, China, would be warmly welcomed. The 8th ACEM will be held in Thailand in 2025. We hope the ACEM 2022 and the great efforts from the scientists in the AAEMS advance the development of environmental research in the Asia-Pacific region and wish success for the next ACEM.

## Data Availability

Not applicable.

## Abbreviations

ACEM: Asian Conference on Environmental Mutagens
AAEMS: Asian Association of Environmental Mutagen Societies
CEMS: Chinese Environmental Mutagen Society
JEMS: The Japanese Environmental Mutagen and Genome Society
QSAR: Quantitative structure-activity relationship
EMS: Environmental mutagen society

